# Preparation and Characterization of Metal–Organic Framework Coatings for Improving Protein Crystallization Screening

**DOI:** 10.3390/nano13142064

**Published:** 2023-07-13

**Authors:** Qin Yang, Zhenkun Zhang, Lin Wang, Xiwen Xing, Jiahai Zhou, Long Li

**Affiliations:** 1 Guangdong Provincial Key Laboratory of Bioengineering Medicine, Department of Cell Biology, College of Life Science and Technology, Jinan University, Guangzhou 510632, China; qin.yang2@siat.ac.cn (Q.Y.); lin.wang5@siat.ac.cn (L.W.); 2 CAS Key Laboratory of Quantitative Engineering Biology, Shenzhen Institute of Synthetic Biology, Shenzhen Institute of Advanced Technology, Chinese Academy of Sciences, Shenzhen 518055, China; zk.zhang@siat.ac.cn

**Keywords:** protein crystallization, crystallization plates, nucleant, metal–organic framework, coatings

## Abstract

Modifying crystallization plates can significantly impact the success rate and quality of protein crystal growth, making it a helpful strategy in protein crystallography. However, appropriate methods for preparing nano-sized particles with a high specific surface area and strategies for applying these nanoparticles to form suitable coatings on crystallization plate surfaces still need to be clarified. Here, we utilized both an ultrasonic crusher and a high-pressure homogenizer to create a nano metal–organic framework (MOF), specifically HKUST-1, and introduced a solvent evaporation method for producing MOF coatings on 96-well crystallization plates to induce protein crystal growth. The morphology of MOF coatings on the resin surface of the plate well was characterized using optical and scanning electron microscopy. Compared to the control group, crystallization screening experiments on nine proteins confirmed the effectiveness of plates with MOF coatings. Applying MOF coatings to crystallization plates is an easy-to-use, time-efficient, and potent tool for initiating crystallization experiments.

## 1. Introduction

Protein crystallization is a critical step in determining the three-dimensional structure of proteins [1,2,3]. However, obtaining well-formed protein crystals suitable for X-ray diffraction presents a significant challenge due to the complex nature of protein molecules, making it difficult for them to pack well in crystallization conditions [4,5]. To address this challenge, researchers use protein crystallization screening to test various conditions, such as protein concentration, buffer type, precipitant, pH, temperature, and additives, to find the optimal conditions [6,7,8]. By screening a wide range of conditions, researchers can increase their chances of finding the best conditions for protein crystallization, leading to the successful determination of a protein’s three-dimensional structure. Consequently, it is essential to develop methods that maximize the screening success rate within the constraints of limited crystallization conditions.

One way to improve the success of the crystallization process is by grafting molecules or self-assembled membranes on the surface of crystallization screen plates [9]. The plate surface can affect protein adsorption, crystal nucleation, growth, and the morphology and quality of the crystals. Therefore, modifying the plate surface can provide tailored structures that promote crystal growth, improve crystal quality, and enhance the crystallization success rate. For instance, Wang et al. [10] found that grafting cyclodextrin and its derivatives onto crystallization plates improved protein crystallization, and this strategy is both labor-efficient and beneficial for automated crystallization screening. However, the effectiveness of this strategy depends on the grafting density of cyclodextrin or its derivatives, making it difficult to determine the optimal thickness for new proteins. Thus, a more convenient method is needed. Gao et al. [11] developed a novel superhydrophobic coating by manipulating the self-assembly of proteins, and they demonstrated that this coating could accelerate and facilitate protein crystallization on the protein-based superhydrophobic surface. This finding suggests that the interfacial material has excellent potential for modulating the nucleation and growth of biomolecules.

Additionally, self-assembled monolayers on gold-coated glass coverslips and surface-localized poly(ethylene glycol) (PEG) and other neutral, hydrophilic polymers have improved protein crystallization via vapor diffusion from drops [12]. Pechkova et al. [13,14,15] developed the Langmuir–Blodgett homologous protein thin film template in 2001, which involved transferring a homologous protein thin film made using Langmuir–Blodgett technology onto a cover glass to modify the traditional hanging drop vapor diffusion method for promoting protein crystallization. The revised form improved crystal quality and increased the crystal growth rate of the model proteins, including lysozyme, thaumatin, proteinase K, and human insulin. Although the methods mentioned above have achieved initial success, they cannot maximize the success rate of crystal screening due to the limited specific surface area produced by the grafting method and self-assembled membranes.

**Figure 1:**
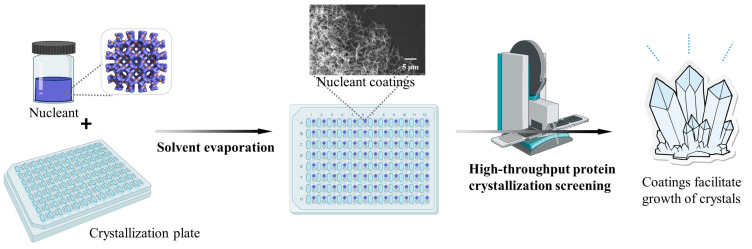
Schematic diagram of the process for using a nucleant-coated 96-well crystallization plate for the high-throughput screening of protein crystals.

Using nanomaterials to create coatings on crystallization plates may be a promising surface modification method for obtaining a more efficient approach than grafting methods and self-assembled membranes, as nanomaterials possess an extremely high surface area. Previous research reported the enhancement of protein crystallization using nano-sized metal–organic frameworks (HKUST-1). By exploring using HKUST-1 nucleant to improve crystal quality, researchers increased the maximum resolution of data from 3.6 Å to 2.3 Å for a challenging protein sample, ergothioneine synthase (EgtB). In addition to improving crystal growth and producing higher-quality crystals, nucleants can enhance the reproducibility of crystallization experiments and enable the use of smaller sample volumes [16,17,18,19,20]. However, the suitable methods for preparing nano-sized HKUST-1 particles and the strategies for applying these nanoparticles to form suitable coatings on the surface of crystallization plates remain unclear. To address this, we adapted treatment using an ultrasonic crusher and a high-pressure homogenizer to create nano-HKUST-1 and coated 96-well crystallization plates using a solvent evaporation method, as illustrated in Figure 1. The main focus of this study was to control the uniformity of the HKUST-1 coating and its distribution on a 96-well surface by adjusting the concentration of nucleant and the volume added to the well. After obtaining an appropriate HKUST-1 coating, the crystallization efficiency of the coating was evaluated using lysozyme, including crystallization success rate, crystallization induction time, and the number and size of crystals. Finally, nine model proteins were used to test the HKUST-1-coated crystallization plates in a crystallization screening experiment, and the resulting crystals were analyzed. Through this approach, we hope to optimize protein crystallization and advance the field of protein crystallography.

## 2. Materials and Methods

### 2.1. Materials

Lysozyme from chicken egg white, sodium acetate and sodium chloride were purchased from Sigma-Aldrich Inc. (St. Louis, MO, USA) Cupric nitrate hemipentahydrate, benzene-1,3,5-tricarboxylic acid, concanavalin A, RNase a, urease, bovine serum albumin, myoglobin, papain, lipase, and cytochrome C were purchased from Aladdin Industrial Inc. (Shanghai, China).

### 2.2. Preparation of HKUST-1 Suspension

HKUST-1 was synthesized following the literature procedure [21]. Dissolving 6.0 g of cupric nitrate hemipentahydrate in 250 mL water, we added 4.0 g ben-zene-1,3,5-tricarboxylic acid to a 250 mL ethanol–water mix (1:1, *v*/*v*), stirring until fully soluble. The solution was moved to a 250 mL teflon-lined autoclave, heated to 110 °C for 18 h to form small crystals, then cooled to room temperature. Blue crystals were then separated by filtration, washed multiple times with an ethanol–water mixture, and dried. To prepare aqueous and ethanol HKUST-1 suspensions, 80 mg of HKUST-1 was separately dissolved in 20 mL of water and anhydrous ethanol, respectively. The suspensions were then treated using an ultrasonic crusher and a high-pressure homogenizer. The JY92-IIN ultrasonic crusher (Scientz, Ningbo, China) operated at 360 W for 30 min, while the UH-06 homogenizer (Union-Biotech Co., Shanghai, China) ran at 600 bar for 5 min. These procedures resulted in four types of HKUST-1 suspensions: water solvent and ultrasound, water solvent and high pressure, ethanol solvent and ultrasound, and ethanol solvent and high pressure. All suspensions were stored at 4 °C in a refrigerator.

### 2.3. Preparation and Characterization of HKUST-1 Coatings

Four different types of HKUST-1 suspensions were prepared at concentrations of 0.5 mg/mL, 1 mg/mL, 2 mg/mL, and 4 mg/mL, respectively. These suspensions were then dropped onto a 96-well crystallization plate in volumes of 0.5 μL, 1 μL, and 2 μL. The plate was subsequently dried in an oven at 65 °C for 60 min, resulting in the formation of HKUST-1 coatings on the plate. The drying process facilitated the formation of HKUST-1 coatings on the plate. The surface morphology of the HKUST-1 coatings was analyzed using a Phenom Pharos G2 scanning electron microscope (SEM) from Thermo Fisher (Waltham, MA, USA). The dried coatings were first sputter-coated with gold for 60 **s** at 8 W and 6 Pa pressure. Subsequently, the samples were analyzed at an accelerating voltage of 15 kV. Powder X-ray diffraction (XRD, Bruker D8 ADVANCE diffractometer at the SAIF, CUSAT, Kochi, Japan, Cu Kα, 5−50° 2θ) was used to explore the structure of the HKUST-1 coatings after different treatments.

### 2.4. Crystallization Experiment

Four different types of HKUST-1 coatings were utilized for protein crystallization. We added 11 mg/mL lysozyme to each type of HKUST-1 coating, with lysozyme without HKUST-1 coatings serving as a control group. Protein crystallization was performed using the sitting-drop vapor-diffusion method on 96-well plates. In all crystallizations, 1.0 M NaCl solution and 0.1 M sodium acetate (pH 4.25) were used as the reservoir solutions. We mixed 1.0 µL of protein solution with 1.0 µL of reservoir solution to set up the crystallization, and each well had a total reservoir solution volume of 50 µL. The crystallization plate was stored at 289 K after setup. Nine model proteins and one HKUST-1 coating (prepared with water solvent and high-pressure treatment) were used for crystallization screening trials. Commercially available 96-condition screens (Crystal Screen 1 and 2, Hampton Research USA) were dispensed. Each protein was mixed with each screen condition in a 1:1 ratio to form a 600 nL drop. Control drops without HKUST-1 coatings were used for comparison. Protein crystals were identified using protein crystal dyes or through single-crystal diffraction experiments.

## 3. Results and Discussion

### 3.1. Preparation and Characterization of HKUST-1 Coatings

**Figure 2:**
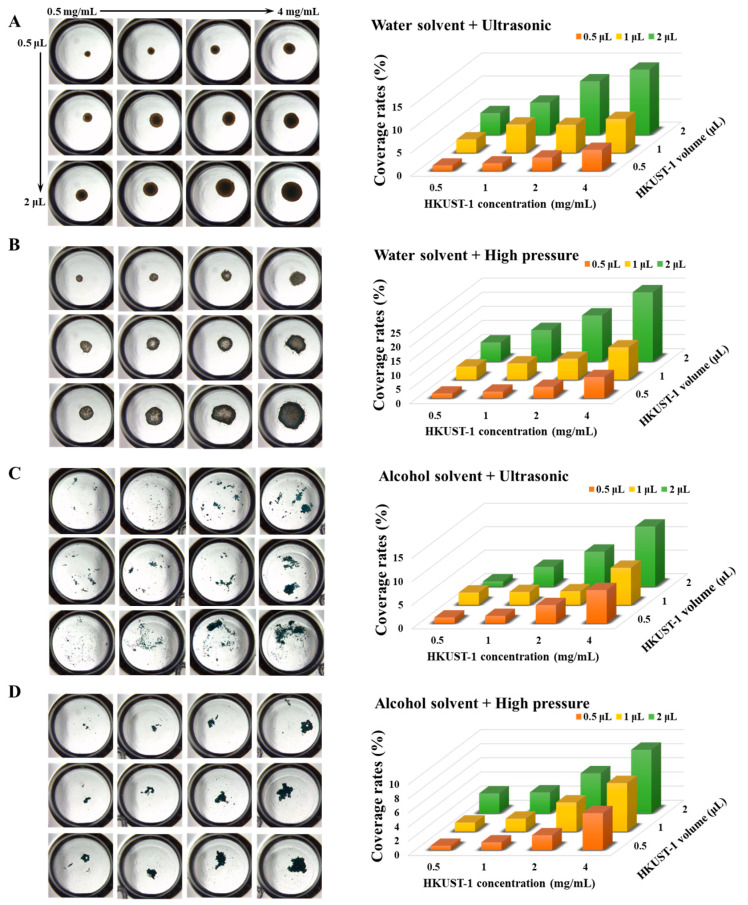
HKUST-1 coatings on 96-well crystallization plates. The left image displays optical microscopy images of HKUST-1 coatings on the plate well. The coatings are arranged in a 4 × 3 screen grid and prepared using different solvents and treatments: water solvent and ultrasound (**A**), water solvent and high pressure (**B**), alcohol solvent and ultrasound (**C**), and alcohol solvent and high pressure (**D**). The right image illustrates the coverage rate of HKUST-1 coatings on the entire well, representing the proportion of the vessel surface area covered by the coatings. HKUST-1 suspensions were prepared at concentrations of 0.5 mg/mL, 1 mg/mL, 2 mg/mL, and 4 mg/mL at volumes of 0.5 μL, 1 μL, and 2 μL added to each well, respectively. Note: The coverage rate in the right image was obtained by integrating the left image using ImageJ software (version 1.8.0). There is a one-to-one correspondence between the images on the left and right.

We initially investigated the impact of nucleant HKUST-1 dosage, treated using an ultrasonic crusher and a high-pressure homogenizer, on the subsequent coating preparation on 96-well plates, as shown on the left side of Figure 2. To regulate the final dosage of the nucleant, we adjusted both the concentration of HKUST-1 and the volume of the HKUST-1 suspension added to the well or vessel. In Figure 2A, as the nucleant’s concentration or the volume of the nucleant suspension added to the well increases, the area covered by the nucleant correspondingly increases. With a concentration of 0.5 mg/mL HKUST-1 and 0.5 μL of the suspension added to the well, the HKUST-1-covered area accounted for 1.2% of the entire vessel area. However, increasing the concentration to 4 mg/mL and adding 2 μL of the suspension led to a 14.1% coverage ratio.

Thus, adjusting the concentration and volume of the nucleant suspension added to the well effectively controls the nucleant coverage. Additionally, we examined the impact of solvents and treatment types used in the HKUST-1 suspension on the subsequent coating preparation on 96-well plates, as shown in Figure 2A–D. Regarding solvents, water’s high surface tension causes nucleant particles to aggregate [22,23], and as water evaporates, the contact area between HKUST-1 and the vessel surface decreases, forming a dotted coating. In contrast, ethanol’s lower surface tension allows HKUST-1 to disperse better, resulting in a dispersed coating after evaporation. In Figure 2A,C, the coverage of the coatings made using both solvents is similar. Still, in Figure 2B,D, water as a solvent obtained a higher coverage of about 24.5% compared to ethanol, which achieved about 8.9% coverage. Therefore, using water as a solvent and high-pressure treatment results in the highest coverage rate of the coating on the plate surface.

**Figure 3:**
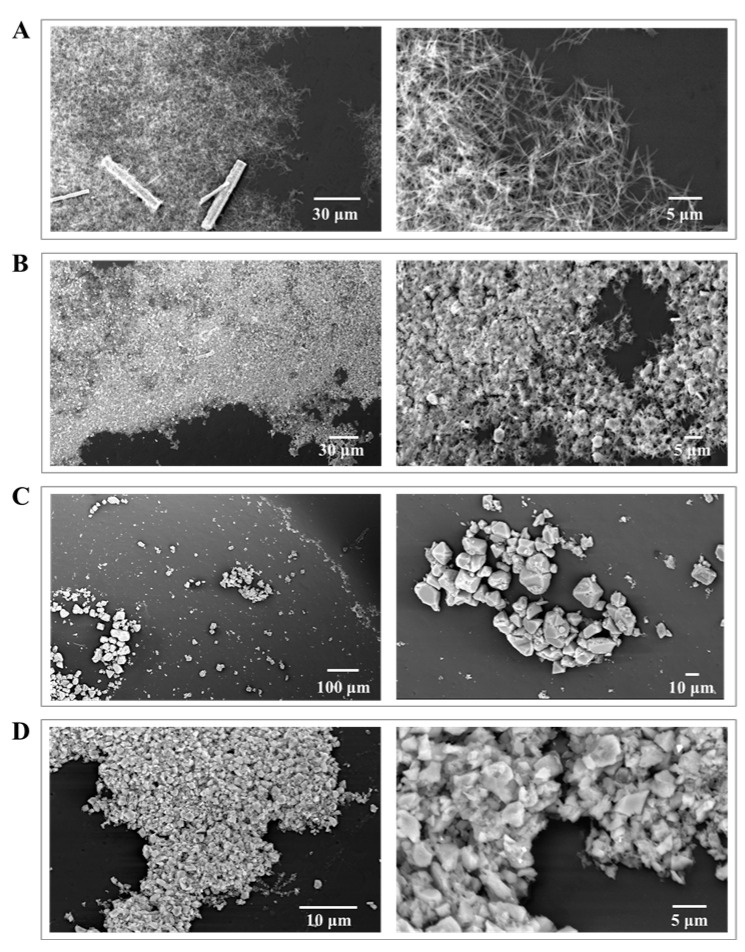
SEM images of HKUST-1 coatings on 96-well crystallization plates obtained using different solvents and treatments: water solvent and ultrasound (**A**), water solvent and high pressure (**B**), alcohol solvent and ultrasound (**C**), and alcohol solvent and high pressure (**D**).

To better understand the coatings, we investigated the morphology, including the shape and size, of HKUST-1 particles of coatings under different solvents and treatments using scanning electron microscopy (SEM). This SEM investigation encompassed the analysis of both shape and size variations. Initially, as-synthesized HKUST-1 particles appeared as tetragonal bipyramidal single crystals. SEM images revealed that using water as the solvent led to smaller particles (Figure 3A,B), while ethanol resulted in larger particles (Figure 3C,D). We further studied the effects of ultrasonic treatment and high-pressure homogenization on the shape and size of HKUST-1 particles. SEM images showed that ultrasonic treatment with ethanol left single crystals visible, while high-pressure homogenization fragmented crystals with particles remaining at the micron level. Using water and high-pressure homogenization, HKUST-1 shattered into nanoparticles, undergoing significant morphological changes. SEM analysis indicated that water as the solvent produced coatings with a larger specific surface area than ethanol due to the complete fragmentation of HKUST-1 into nanoparticles, providing a higher surface area for the coating [24]. As seen in Figure S1, the XRD results demonstrate a consistent preservation of HKUST-1’s structure when ethanol is used as a solvent, irrespective of the type of treatment methods used. In contrast, for the HKUST-1 samples treated with water as a solvent and ultrasound, we have identified potential structural modifications, which could be attributed to the ultrasound treatment. Based on these observations, we have excluded this combination of treatment methods from our future experiments.

### 3.2. Effectiveness of Different HKUST-1 Coatings on Protein Crystallization

**Figure 4:**
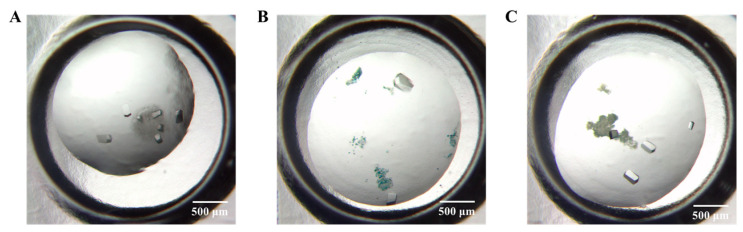
Lysozyme crystallization is facilitated by three types of HKUST-1 coatings at 11 mg/mL lysozyme, 0.1 M sodium acetate (pH 4.25), and 1 M NaCl solution. There are three types of HKUST-1 coatings: water solvent and high pressure (**A**), alcohol solvent and ultrasound (**B**), and alcohol solvent and high pressure (**C**).

After characterizing the four types of coatings, we proceeded with protein crystallization studies using HKUST-1 coatings formed under conditions of the same concentration (4 mg/mL) and volume (0.5 uL), as this condition resulted in ideal coating coverage. Due to the structural changes observed in HKUST-1 samples treated with water as a solvent and ultrasound, as indicated by the XRD results, we will only consider the effects of the other three treatment methods on protein crystallization for the subsequent analysis. Finally, using the model protein lysozyme, we examined the impact of the three coatings on lysozyme crystallization. The results show that crystals appeared in all three coatings, but water-based coatings induced the formation of more crystals in a more concentrated manner (Figure 4A). In contrast, the crystals produced by the ethanol-based coatings are more dispersed (Figure 4B,C). In Figure 4, we observed that among the three coatings tested for lysozyme crystallization, the one treated with water solvent and high pressure induced the most crystal formation, with crystals growing around the coating area. In contrast, while the ethanol-treated coating were able to form crystals, their quantity was significantly lower than that with the water solvent and high pressure-treated coating. Therefore, we chose the water solvent- and high pressure-treated coatings for further research.

**Figure 5:**
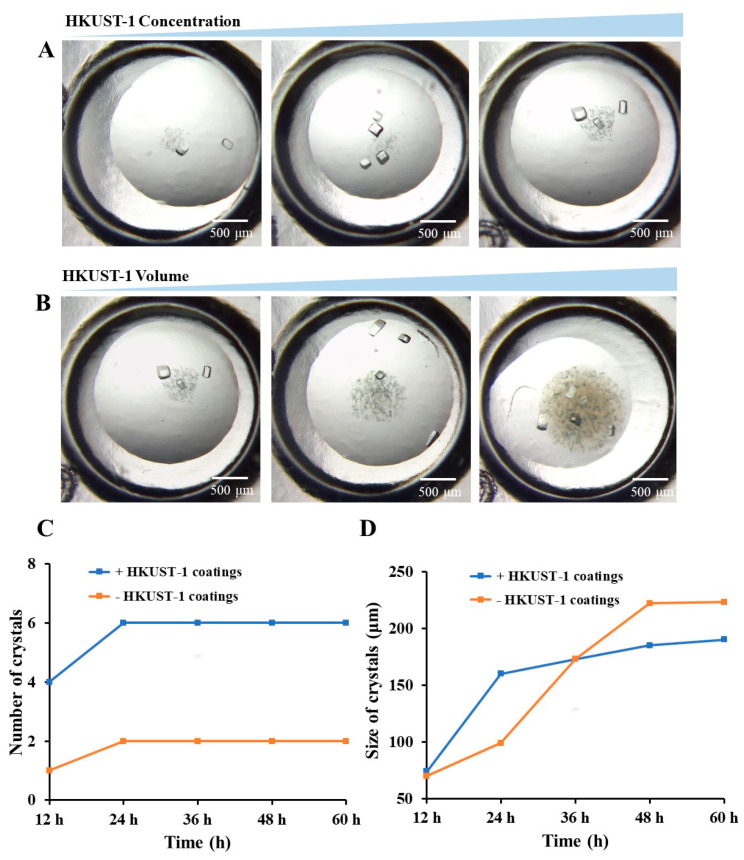
The crystallization of lysozyme on coatings prepared with HKUST-1 aqueous suspensions of different concentrations (**A**) and volumes (**B**). The changes in crystal quantity (**C**) and crystal size over time with or without HKUST-1 coatings (**D**). Note: For ease of estimation, the longest edge of the crystal has been used as the size of crystals (µm).

Moreover, we investigated the impact of HKUST-1 coating dosage on protein crystallization by varying the concentration and volume of the HKUST-1 suspension. As the concentration and volume increased, the coating coverage within the plate well expanded, promoting protein crystal nucleation and growth around the coating. This change led to a rise in the total number of lysozyme crystals (Figure 5A,B) without notable changes in crystal morphology. Then, when the concentration was 1 mg/mL and the volume was 0.5 μL, the quantity and size of crystals within 60 h were summarized for samples with and without HKUST-1 coatings (Figure 5C,D). Results indicated that the crystal count doubled with HKUST-1 coatings. Regarding size, lysozyme crystals induced by HKUST-1 coatings were initially larger within the first 24 h but were later surpassed by crystals without HKUST-1 coatings. This phenomenon might be due to the nucleant’s induction effect, initially facilitating faster crystal growth and larger sizes. However, with a threefold increase in crystals formed using HKUST-1 and limited protein in the solution, crystal growth slowed.

**Figure 6:**
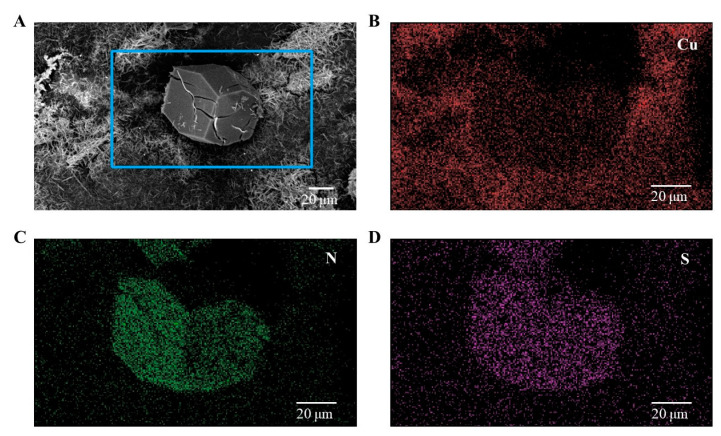
SEM images of protein crystal growth on the surface of the HKUST-1 coating (**A**). EDS mapping images showing the chemical distributions of copper (**B**), nitrogen (**C**), and sulfur (**D**).

To provide evidence that protein crystals grow on the surface of the nucleant coating, we executed an analysis that mainly involved identifying distinct nitrogen (N) and sulfur (S) elements within the protein crystals and the unique copper (Cu) element in HKUST-1. Given that the nucleating agent coating did not entirely encompass the area within the perforated plate, we observed the concurrent presence of regions with N, S, and Cu elements. This initial observation allowed us to infer that protein crystal growth likely occurs on the surface of the nucleant, though further investigations would be required to substantiate this assumption and provide a more rigorous confirmation. Energy dispersive spectroscopy (EDS) mapping was used to determine the elemental composition and distribution of protein crystals grown on the HKUST-1 coating surface. Figure 6A presents SEM images of protein crystal growth on the HKUST-1 coating surface, while EDS mapping images display the chemical distributions of copper (Figure 6B), nitrogen (Figure 6C), and sulfur (Figure 6D). The EDS mapping images revealed that copper atoms were dispersed throughout the HKUST-1 coating, whereas nitrogen and sulfur atoms were concentrated in the protein crystals. This information offers insights into the interaction between the protein crystals and the HKUST-1 coating.

### 3.3. The Application of HKUST-1 Coatings for Crystals Screening Experiments

In light of the experimental findings, utilizing water as a solvent in conjunction with high-pressure treatment exhibited superior stability and efficacy in promoting protein crystallization. As a result, we performed comprehensive protein crystallization screening experiments employing coatings prepared with this optimal combination. We screened nine proteins with and without HKUST-1 coatings using Crystal Screen and Crystal Screen 2 reagent kits, chosen for their diverse crystallization conditions and widespread use. We also employed a pre-crystallization test kit to determine optimal protein concentrations before starting the experiment. Results in Table 1 show that HKUST-1 coatings increased crystal hits for most proteins, with thirteen new hits for lysozyme, seven for concanavalin A, five for urease, two for lipase, and two each for RNase A and papain.

**Figure 7:**
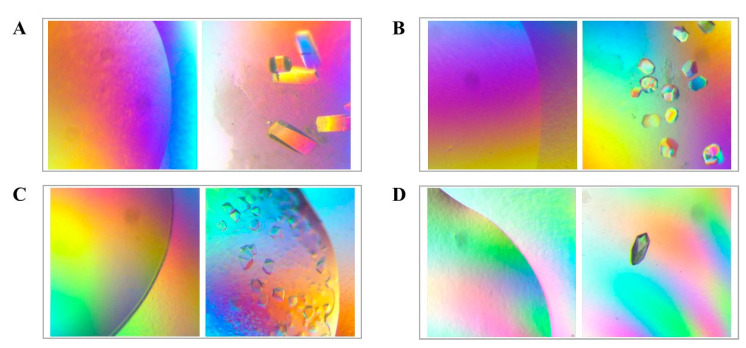
Optical microscope images of partial new hits during initial screening by using HKUST-1 coatings. (**A**) Lysozyme, (**B**) concanavalin A, (**C**) RNase A, and (**D**) papain, In the gray rectangle frame, the left picture shows the group without HKUST-1 coatings and the right picture shows the group with HKUST-1 coatings. The scale bar represents 100 μm.

Interestingly, no new hits were observed for myoglobin and cytochrome C, and bovine serum albumin (BSA) produced no crystals in either condition. Notably, proteins such as myoglobin, papain, and lipase did not crystallize at low concentrations, likely due to low supersaturation [25]. Yet, HKUST-1 coatings led to the discovery of new crystallization conditions for these proteins, suggesting their potential usefulness for challenging-to-crystallize proteins. However, concanavalin A and BSA showed inhibitory effects with HKUST-1 coatings. This may be due to phase separation on the coating’s surface at high supersaturation, causing oil or precipitate formation and inhibiting crystal growth [26,27]. Our preliminary crystal screening experiments demonstrated HKUST-1 coatings’ effectiveness in discovering new crystallization conditions (Figure 7). These results suggest that HKUST-1 coatings can enhance protein crystallization and identify new conditions, but their impact may vary depending on the protein. Further investigation is needed to fully understand HKUST-1 coatings’ effects on protein crystallization.

**Figure 8:**
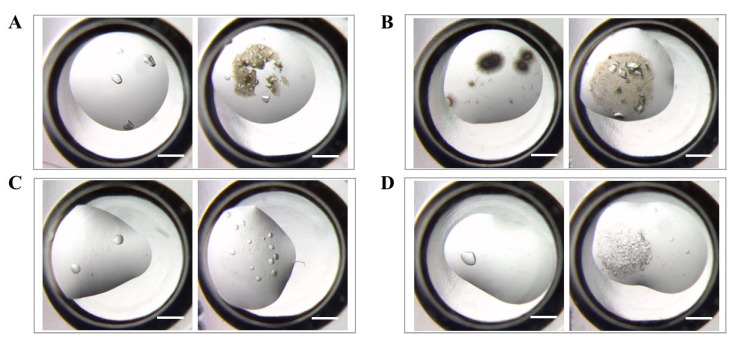
Optical microscope images of lysozyme crystallization at E1 (**A**) and E9 (**B**) and concanavalin A crystallization at F3 (**C**) and D11 (**D**) in the Hampton Crystal Screen kit. The left picture shows the group without HKUST-1 coatings, and the right picture shows the group with HKUST-1 coatings. The scale bar represents 500 μm.

Figure 8 serves as an illustrative example of the coating’s effect on protein crystal number and shape. Without the coating, lysozyme forms only one morphology, such as needle-shaped or single crystals (Figure 8A,B). However, with the addition of the nucleant coating, both classic tetragonal crystals and needle-shaped or single crystals are promoted. Intriguingly, besides the classic tetragonal crystals, a substantial number of needle-shaped crystals also cover the coating’s surface, resulting in a darker overall color (Figure 8B). Moreover, the impact of HKUST-1 coatings on crystal formation extends beyond lysozyme, as demonstrated by the significant increase in concanavalin A crystal numbers observed with the coating (Figure 8C,D). Overall, these findings suggest that HKUST-1 coatings facilitate the identification of new hits during crystal screening and optimize protein crystallization conditions for improved crystal quality and yield.

## 4. Conclusions

In conclusion, this study presents a solvent evaporation method for producing MOF coatings on 96-well crystallization plates to improve protein crystallization. We optimized the particle packing density and dispersion on the vessel surface by controlling the nucleant concentration and volume. We examined the impact of an ultrasonic crusher and a high-pressure homogenizer on the HKUST-1 suspension, revealing that the high-pressure homogenizer treatment provided better dispersion and increased the specific surface area of the coatings. These HKUST-1 coatings significantly enhanced the effectiveness of protein crystallization screening experiments compared to the control group. The method offers an easy-to-use, time-efficient, and powerful tool for structural biologists initiating crystallization experiments. Further research is needed to understand the mechanism of action of HKUST-1 coatings in protein crystallization and optimize their application. Overall, this study sheds light on the potential of surface coatings to augment the success rate of protein crystallization, contributing to the development of effective strategies in protein crystallography.

## Figures and Tables

**Table 1:** Crystallization screening hits for nine proteins using 96-well plates in the presence and absence of HKUST-1 coatings.

Protein	Concentration (mg/mL)	− Coatings	+ Coatings		
			Total	New	Lost
Lysozyme	6.5	2	15	13	0
Concanavalin A	4.5	11	13	7	5
RNase A	30	2	4	2	0
Urease	1	3	8	5	0
Bovine serum albumin	35	1	0	0	1
Myoglobin	5	0	1	1	0
Papain	1	0	2	2	0
Lipase	4	0	2	2	0
Cytochrome C	1.5	0	0	0	0

Note: To prepare protein samples, a buffer of 20 mM Tris-HCl, pH 7.0, was selected for concanavalin A, RNase A, urease, bovine serum albumin, myoglobin, papain, lipase, and cytochrome C. Lysozyme was prepared using water.

## Data Availability

The data presented in this study are available upon request from the corresponding author.

## Supplementary Materials

The following supporting information can be downloaded at: https://www.mdpi.com/article/10.3390/nano13142064/s1, Figure S1: XRD of HKUST-1 coatings using different solvents and treatments.
