# Supplementary material for: Preparation and Characterization of Metal–Organic Framework Coatings for Improving Protein Crystallization Screening
Source: Nanomaterials (Basel). 2023 Jul 13;13(14):2064. doi: 10.3390/nano13142064 (PMC10386356; doi:10.3390/nano13142064)
Supplement: Supplementary file 1 [file nanomaterials-13-02064-s001.zip › nanomaterials-2448350-supplementary.pdf]

Supplementary Material

Preparation and Characterization of Metal–Organic Framework Coatings for Improving Protein Crystallization Screening

Qin Yang [1,2], Zhenkun Zhang [2], Lin Wang [1], Xiwen Xing [1,*] and Jiahai Zhou [2,*], Long Li [2,*]

[1] Guangdong Provincial Key Laboratory of Bioengineering Medicine, Department of Cell Biology, College of Life Science and Technology, Jinan University, Guangzhou, China; xingxiwen0025@sina.com (X.X.)

[2] CAS Key Laboratory of Quantitative Engineering Biology, Shenzhen Institute of Synthetic Biology, Shenzhen Institute of Advanced Technology, Chinese Academy of Sciences, Shenzhen, 518055 China; jiahai@siat.ac.cn (J.Z.); long.li@siat.ac.cn (L.L.)

[*]Correspondence: long.li@siat.ac.cn; jiahai@siat.ac.cn; xingxiwen0025@sina.com

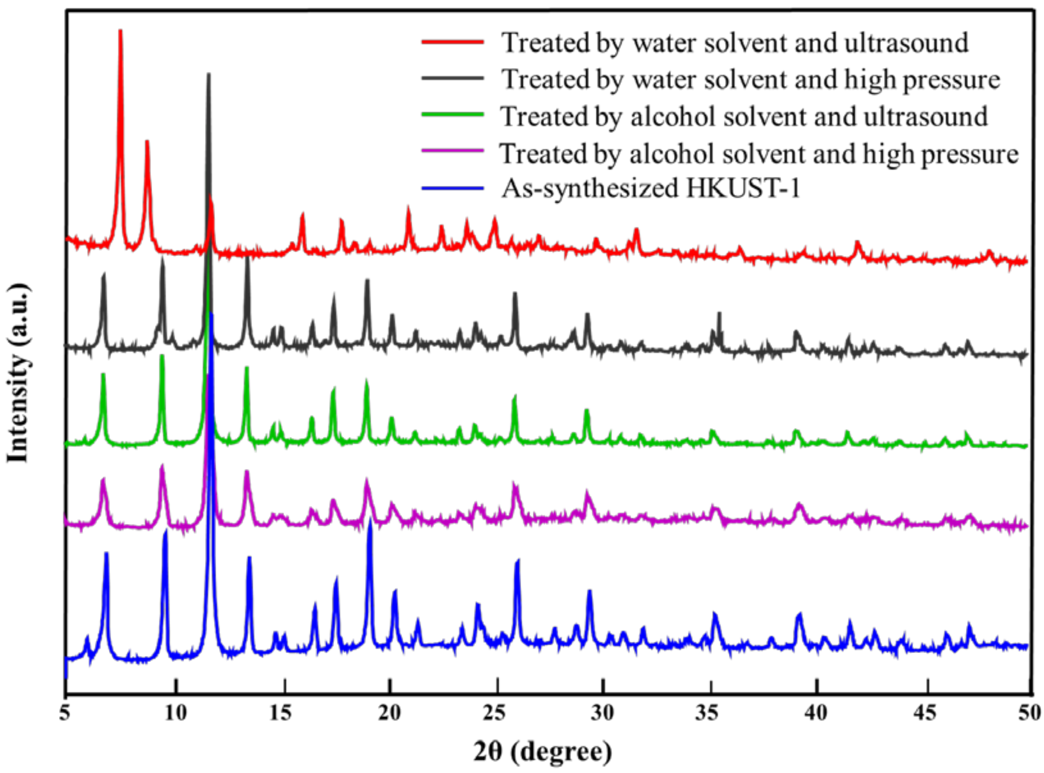

Figure S1: XRD of HKUST-1 coatings using different solvents and treatments. Apart from HKUST-1 treated with water solvent and ultrasound (red line), the positions of the diffraction peaks of other treated HKUST-1 are consistent with the literature reported in the *International Journal of Hydrogen Energy*, 2012, 37(18), 13865-13871.
